# Model-based cost-minimization analysis of electrochemical hydrogen peroxide screening for oxidative stress evaluation using airways epithelial cell systems

**DOI:** 10.1007/s44164-026-00146-6

**Published:** 2026-08-05

**Authors:** Daniele Donzì, Maria Rita Marabeti, Paola Pinto, Anna Bonomolo, Serena Di Vincenzo, Maria Ferraro, Caterina Cascio, Giuseppina Chiappara, Chiara Cipollina, Nadia Moukri, Bernardo Patella, Andreina Bruno, Rosalinda Inguanta, Elisabetta Pace

**Affiliations:** 1 Institute of Translational Pharmacology, Italian National Research Council (CNR), 90146 Palermo, Italy; 2 PhD National Program in One Health approaches to infectious diseases and life science research, Department of Public Health, Experimental and Forensic Medicine, University of Pavia, 27100 Pavia, Italy; 3 Fondazione Ri.MED, 90100 Palermo, Italy; 4 Dipartimento di Ingegneria, Università degli Studi di Palermo, 90100 Palermo, Italy

**Keywords:** Economic analysis, Research costs, Electrochemical sensors, Bronchial epithelial cells, Cigarette Smoke Extract (CSE), Oxidative stress.

## Abstract

Oxidative stress is a common event in a variety of chronic diseases and its detection is confined to equipped laboratories with dedicated instruments and with personnel with high specialization. Electrochemical sensors have been demonstrated to be effective, easy-to-use, and reliable tools for quantifying extracellular hydrogen peroxide (H₂O₂) in airway epithelial preclinical models. Economic analyses of biomedical studies are increasingly needed. The present cost-minimization analysis compares the experimental costs of a traditional oxidative stress workflow with those of a screening strategy based on electrochemical H₂O₂ detection. The experimental design included bronchial epithelial cells exposed to cigarette smoke extract (CSE), as oxidative stress inducer, at three different concentrations and for three time points. As downstream events to CSE exposure, it was planned to assess mitochondrial superoxide production, toll-like receptor 4 (TLR4) and NF-kB expression, IL-8 gene expression and concentrations, and fluorescent advanced glycation end products (fAGE). The detection of H₂O₂ by electrochemical sensors in culture supernatants enables downstream events to be assessed only under conditions in which an extracellular oxidative response is detected, allowing the identification of the optimal time point and stimulus concentration. Considering a failure rate of 23% for both methods, the sensor-based approach reduces the overall experimental cost by 85.67% compared with the traditional ones. This value reflects the decrease in consumables and personnel time associated with preliminary screening performed with electrochemical sensors. In conclusion, the present analysis supports the utility of electrochemical sensors in optimizing research costs of preclinical studies aimed at assessing oxidative homeostasis perturbation and relative downstream events.

## Key points

Electrochemical sensors are cheap and reliable tools for assessing oxidative stress in real-time and in situ analysis using small and portable systems.

This innovative approach has been demonstrated effective to detect oxidative stress on airway epithelial preclinical models.

Herein, it is provided evidence that the electrochemical sensor-based screening stands out as an effective and economical strategy for oxidative stress studies, offering early identification of pertinent samples and reducing the burden of downstream analyses thus optimizing research costs.

## Introduction

Oxidative stress is the result of an excessive production of reactive oxygen species (ROS) or of reactive nitrogen species (RNS) overcoming antioxidant mechanisms [1]. Increased oxidative stress promotes the modification of proteins, lipids and nucleic acids in body fluids and in the cells. The uncontrolled release of ROS within the lung amplifies tissue inflammation, structural damage, accelerates tissue remodeling, and alters/reduces the response to drugs such as corticosteroids [2, 3]. Increased oxidative stress is a crucial mechanism in the pathogenesis of several chronic inflammatory degenerative diseases, including lung diseases [1–4]. The ageing population is associated with an increased incidence and prevalence of chronic diseases, including chronic lung diseases [5]. The increasing social and healthcare costs associated with chronic disease management have encouraged the development of research strategies aimed at preventing and reducing disease progression. The assessment of airways oxidative homeostasis may provide an efficient tool for monitoring disease onset, progression and response to therapy [6–8]. During the last years, several efforts have been directed toward the optimization of preclinical cellular models to reproduce a specific pathological model for studying molecular mechanisms relevant in disease pathogenesis and for testing drugs [9–12]. In this regard, several studies have demonstrated the utility of using bronchial epithelial cells injured with cigarette smoke extract (CSE) to dissect some molecular events associated with pathogenesis and therapies of Chronic Obstructive Pulmonary Disease (COPD) [13, 14]. However, the maintenance of preclinical cellular models per se and the assessment of cellular and molecular events related to oxidative stress and promoted by external stimulation are expensive, in terms of instruments and specialized personnel (or human capital) and time consuming and there is an increasing need to control their cost [15]. Technological innovation can provide simple and reliable methods for the direct assessment of oxidative stress thus promoting new advances in precision/personalized medicine in a sustainable way. In this regard, previous studies used electrochemical sensors to quantify extracellular H₂O₂ in cell supernatants from airway models [16–19]. These studies have allowed us to demonstrate that the developed electrochemical sensors are characterized by simplicity, reliability, low cost, excellent performance in terms of sensitivity, selectivity, limit of detection (LOD), and rapidity of response. The results obtained with the electrochemical sensors developed by our group are comparable to those obtained with a cytofluorometer or spectrofluorometer [16–19]. The sensor properties described above therefore enable reliable measurement of extracellular H₂O₂ as a screening marker of oxidative stress, even in laboratories without expensive equipment and highly specialized personnel. The COVID-19 pandemic and the global economic slowdown have led to reduced budgets globally. Supply chain disruptions and rising energy prices have led to increases in both consumables and scientific equipment. As a result, inflation has led to a substantial increase in research expenditures against a modest increase in research grants. Therefore, laboratory financial management has never been more important. To the best of our knowledge, no previous study has evaluated the economic impact of integrating electrochemical sensors as a screening tool to optimize oxidative stress studies. Based on this, the present model-based cost-minimization analysis provides, for the first time, evidence on the utility of a cheap, homemade electrochemical sensor to perform oxidative stress studies in bronchial epithelial cell cultures, optimizing direct research costs. Sensitivity, accuracy, validation, performance, and comparison with other established oxidative stress detection techniques of the electrochemical sensor of the present study were all previously assessed in previous our studies [17, 20].

## Materials and methods

### Study design

**Fig. 1:**
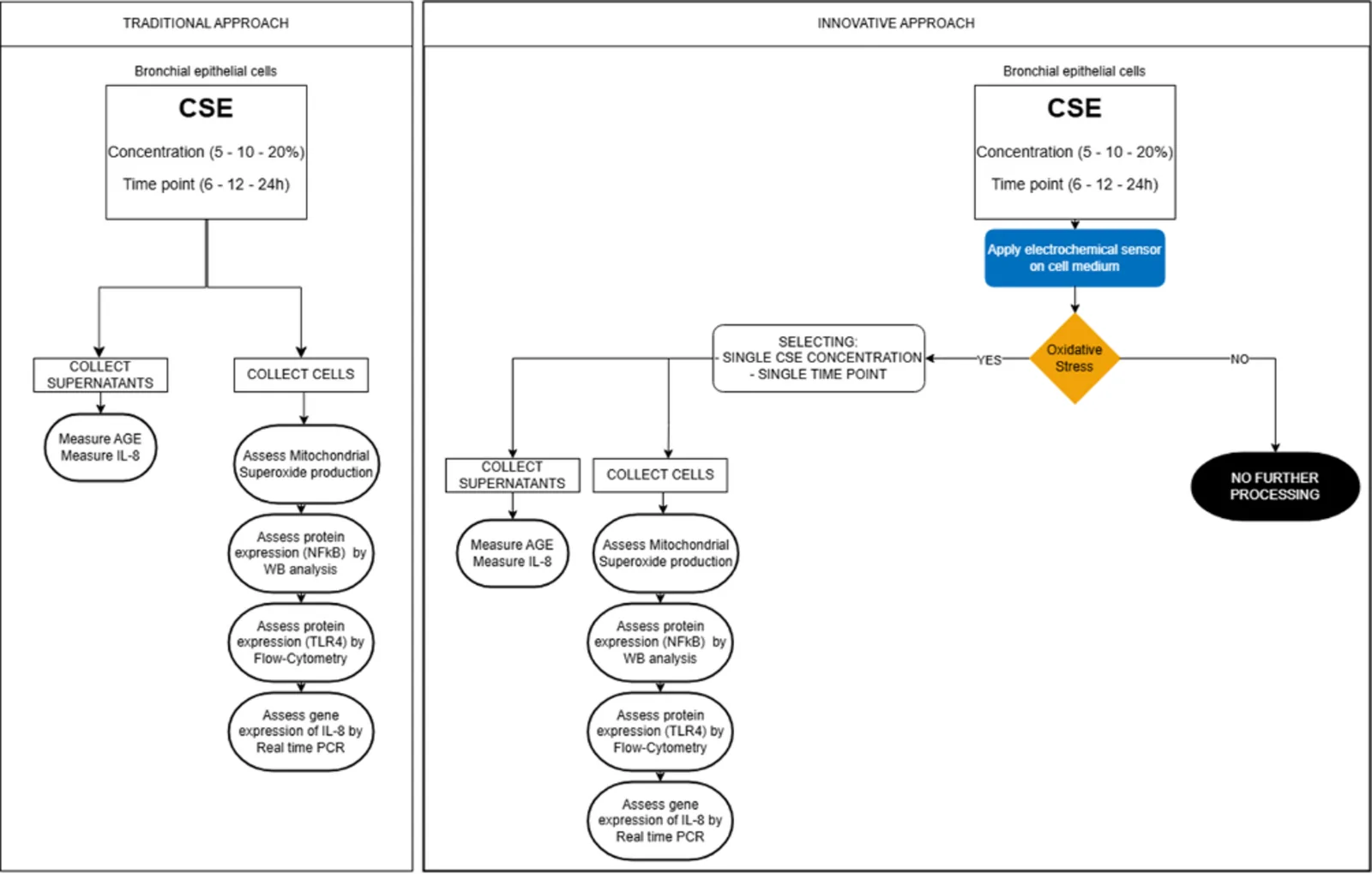
Flow chart of traditional and innovative methods

The design of this model-based cost-minimization analysis is described in Fig. 1 where the traditional and innovative approach flow charts are shown.

In the traditional workflow, bronchial epithelial cells (16HBE) are considered to be exposed to CSE at three concentrations (5%, 10%, and 20%) for 6, 12, and 18 h, after which cells and supernatants are collected to assess total or mitochondrial ROS production by flow cytometry [21], protein expression by Flow cytometry, pro-inflammatory transcription factor assessment by Western blot analysis, gene expression evaluation by Real Time PCR and pro-inflammatory cytokine release by ELISA as previously described with some modifications [12] or oxidative stress marker assessment [14]. In the arm of the new approach, cells are exposed to the three CSE concentrations at the three time points above mentioned and a small aliquot of cell culture supernatant (250 µl) is collected without perturbing cell culture and screened with the electrochemical *sensor*. Only conditions showing an extracellular H₂O₂ response proceed to downstream analyses. The sensor therefore does not measure H₂O₂ at the single-cell surface, but rather quantifies H₂O₂ released by the cells and accumulated in the extracellular culture medium. The cost analysis of the traditional approach, including the % of failure, was performed by a retrospective evaluation of data from an oxidative stress study performed by our group [10]. To quantify the failure rate of traditional oxidative stress workflows, we re-evaluated the dataset previously published by our group [10]. In that study, oxidative stress induction was assessed by ROS production using flow cytometry, and experiments in which the positive control (cigarette smoke extract, CSE) did not elicit ROS increase were classified as failed. The failure rate was calculated as:

$$\%\;\mathrm{Failure}=\left(\mathrm{Failed}\;\mathrm{Experiments}/\mathrm{Total}\:\mathrm{Experiments}\right)\times100=23\%$$

This value was used as a reference to estimate the inefficiency of traditional workflows.

The details of experimental procedures considered to compare the effective costs for oxidative stress putative study are here described below.

### 16HBE culture and treatment

The human bronchial epithelial cell line 16HBE, a human bronchial epithelial cell line obtained from a 1-year-old male and immortalized with SV40 plasmid that retains the differentiated morphology and function of normal airway epithelial cells, is used in this study [22]. 16HBE cells are maintained in a humidified atmosphere of 5% CO_2_ in air at 37 °C and are cultured as adherent monolayers. Eagle’s minimum essential medium (MEM), supplemented with 10% heat-inactivated (56 °C, 30 min) fetal bovine serum (FBS), 1% MEM (non-essential aminoacids), 2 mM L-glutamine and 0.5% gentamicin is used for culturing the cells. For the experiments, 16HBE are seeded and stimulated when they reach the confluence, with CSE at the different concentrations (5%, 10%, and 20%) for 6, 12 and 18 h. Cells and supernatants from samples with induced oxidative stress are further processed by ELISA, Real Time PCR, Western blot analysis and Flow cytometry. Electrochemical *sensor*, applied on small (250 µl) aliquots of cell medium from NT (not treated) and CSE stimulated cells, is used for assessing extracellular H₂O₂.

### Preparation of CSE

CSE is obtained using a peristaltic pump Watson-Marlow 323 E/D (Rotterdam, The Netherlands) using two Kentucky 2R4F research-reference cigarettes (The Tobacco Research Institute, University of Kentucky, USA) without filter. Briefly, each cigarette is smoked for 5 min in 10 mL of PBS to generate a CSE-PBS solution. The CSE solution is then filtered using a 0.22 μm pore filter to remove bacteria and large particles. This solution is considered 100% concentration and then was diluted to the selected concentration [7, 20].

### Flow cytometry for the measure of mitochondrial superoxide production (MitoSOX)

To evaluate the production of mitochondrial superoxide is used the MitoSOX™ Red mitochondrial superoxide indicator (Molecular Probes Waltham, MA, USA). After optimal time of stimulation, the cells are harvested, washed with PBS and stained with 3 µM MitoSOX Red probe for 15 min at 37 °C. Then, 16HBE are washed twice in PBS and then analyzed by flow cytometry using CytoFLEX (Beckman Coulter, Lakeview Pkwy S Drive, Indianapolis, USA). The results are expressed as percentage of positive cells [7, 20].

### Measure of extracellular hydrogen peroxide by electrochemical sensor

Extracellular H₂O₂ is quantified using an electrochemical sensor based on silver as the active material, which is recovered from recycled compact discs (CDs) [17, 20]. The process involved carefully peeling off the silver layer from the surface of the CDs using a double tape as described in our previous work [17]. This silver layer is then used to construct the electrodes for the sensor. A three-electrode configuration is designed using a laser cutter. The sensor is precisely cut into three distinct zones, including one for the deposition of silver chloride (AgCl) paste, one left uncoated, and one for the deposition of graphite paste. The sensors consist of a working electrode (WE) made from un-modified silver layer, a reference electrode (RE area coated with Ag/AgCl paste), and a counter electrode (CE area coated with graphite paste). The deposition of the pastes onto the different areas of the sensor is performed manually, ensuring meticulous attention to detail. The sensors undergo a thermal treatment in an oven at 50 °C for 30 min for paste sintering to enhance adhesion and stability. The fabricated sensor is then fixed onto a flexible acetate sheet, and external contacts are made using copper tape. Finally, the reading area is isolated using adhesive tape to avoid interference from the copper electric contacts.

The obtained sensor is connected to a potentiostat (Palmsens EmStat3, Houten, The Netherlands). Linear Sweep Voltammetry (LSV) is used as the detection technique in MEM medium solution containing different concentrations of H₂O₂. The sensor was also used to quantify H₂O₂ released by 16HBE in the culture medium. The sensor is placed in a homemade cell with a volume of 1 mL. Before sample analysis, a calibration curve is generated for each sensor by measuring known H₂O₂ concentrations in MEM across a voltage scan from − 0.2 V to − 1.0 V. The resulting curve is then applied to all samples analysed with that sensor during the same analytical session. Calibration is therefore performed for each individual sensor rather than for the fabrication batch as a whole.

The analytical repeatability and reproducibility of the electrochemical sensor used in this study has been extensively validated in our previous publications [17, 20], where its performance showed strong agreement with established analytical techniques. The sensors repeatability was assessed in terms of Intra-Assay coefficient of variation (CV) % and reproducibility as Inter-Assay CV%. Taking multiple measurements under identical experimental conditions in the same sensor, Intra-Assay CV% was ≤ 5%. Inter Assay CV%, evaluating measurements of the same experimental conditions across different sensor replicates, was ≤ 5%.

Sensors stored individually with silica gel at room temperature maintained stable baseline current and calibration slope for at least 90 days. Repeated calibration curves confirmed that analytical performance remained within ± 5% of initial values, demonstrating good stability after prolonged ambient storage.

The sensor specifically detects H₂O₂ and does not directly quantify other reactive species, such as superoxide, nitric oxide, or peroxynitrite, which require complementary analytical methods.

### fAGE assessment

Quantitative fluorescence spectroscopy analysis is used to measure fluorescent advanced glycation end products (fAGEs) concentration in 16HBE supernatants [14]. Cell supernatants are centrifuged at 14,000 g for 1 h and then diluted 50-fold with PBS. The fluorescence intensity is recorded at an emission wavelength of 440 nm after an excitation at 350 nm using GloMax^®^ Discover Microplate Reader (Promega Corporation, Madison, Wisconsin, USA). PBS solution is used as blank. The data are expressed in arbitrary units (AU) and normalized to the total protein amount (AU/mg). The total protein is measured by the Bradford assay (Biorad, Hercules, CA, USA). Each sample is analyzed in triplicate.

### Flow cytometry for the assessment of TLR4 expression

To evaluate the expression of TLR4, cells are permeabilized by two washes with permeabilization buffer (PBS with 1% FBS, 0.3% saponin, and 0.1% sodium azide) followed by incubation with specific rabbit PE conjugated polyclonal antibody anti-human TLR4 (eBioscence, San Diego CA) 30 min at the dark and then evaluated by flow cytometry using CytoFLEX (Beckman Coulter, Lakeview Pkwy S Drive, Indianapolis, USA). Negative controls are performed using rabbit immunoglobulins (Dako). Data are expressed as geomean fluorescence intensity or as percentage of positive cells.

### Western blot analysis of NF-kB expression

16HBE pellets are collected upon CSE treatment for 6, 12 and 18 h, and cytoplasmic and nuclear proteins are extracted using a commercial kit (Nuclear and Cytoplasmic Extraction Kit from Thermo Scientific (Waltham, MA-USA). Protein concentrations are evaluated using the Bradford assay (Biorad, Hercules, CA, USA). 30 µg of lysates are resolved on a 4–15% Mini-PROTEAN TGX Precast Protein Gels, then transferred to nitrocellulose membrane (Biorad, Hercules, CA, USA) [20]. The membranes are blocked with Blocking Buffer and probed with NF-kB antibody (8242 S; Cell signaling, Danvers, MA, USA). The membranes are then incubated with IRDye^®^ 680RD Goat anti-Rabbit (LI-COR Biosciences, Lincoln, Nebraska USA). Protein signal is then detected using the Odyssey^®^ Classic Imaging System (LI-COR) and Image Studio™ Software (LI-COR). For loading control, membranes were re-tested with a mouse anti-β-Actin (1:5000; Santa Cruz Biotechnology). Contrast and brightness are adjusted equally across entire images to best visualise protein bands [23]. Analysis densitometric is performed by using Image J program.

### Gene expression by real time PCR

Real-time polymerase chain reaction (PCR) is performed as previously described [7]. 16HBE are stimulated with CSE for 6, 12 and 18 h and the whole RNA is isolated using TRIzol Reagent (Life Technologies, Thermo Fisher Scientific, MA, USA) following the manufacturer’s instruction. 1 µg of RNA is reverse transcribed to cDNA, using iScript cDNA Synthesis kit (Biorad, CA, USA). IL-8 gene expression is evaluated by qRT-PCR conducted by QuantStudio™ 3 Real-Time PCR System (Thermo Fisher Scientific, Waltham, MA, United States)) using specific FAM‐labeled probe and primers (prevalidated TaqMan Gene expression assay for IL-8, Hs00174103_m1; Thermo Fisher Scientific) as previously described [24, 25]. Gene expression is normalized to GAPDH (prevalidated TaqMan Gene expression assay for GAPDH, Hs03929097_g1) as endogenous control gene. The relative quantification of mRNA is obtained with the comparative Ct method (2^ − ΔΔCt) and is plotted as respective fold‐change. Untreated cells (NT) were used as reference sample.

### Evaluation of IL-8 release

The concentrations of the IL-8 in cell supernatants are assessed by commercially available enzyme-linked immunosorbent assays (ELISA) (DuoSet R&D Systems, Minneapolis, MN, USA) following manufacturer’s instructions [21].

### Study disclaimer

This manuscript presents a putative, model-based cost-minimization analysis that combines previously published experimental data with newly calculated economic metrics. No additional cell-culture experiments, animal work, or patient interventions were performed specifically for this article. All biological results are drawn from peer-reviewed sources cited in the Methods section; therefore, no new ethical approvals or cell-line authentications were required. The primary objective is to illustrate how an early, low-cost electrochemical screening step can reduce laboratory expenditure under realistic, literature-derived conditions rather than generate new wet-lab data.

The base-case model assumes a proportional reduction in condition-dependent personnel, consumable, and reagent costs when the number of analysed time points and CSE concentrations are reduced. Fixed components, including shared controls, calibration standards, and bulk reagent preparation, were not modelled separately and may not decrease proportionally. To assess the robustness of the economic results, a sensitivity analysis was performed by applying a hypothetical 10% technical sensor failure rate to the failure-adjusted screening cost, separately from the 23% biological non-response rate derived from the previously published dataset.

## Results

We initially assessed the cost of a putative study approached by the traditional method illustrated in the Fig. 1.

**Fig. 2:**
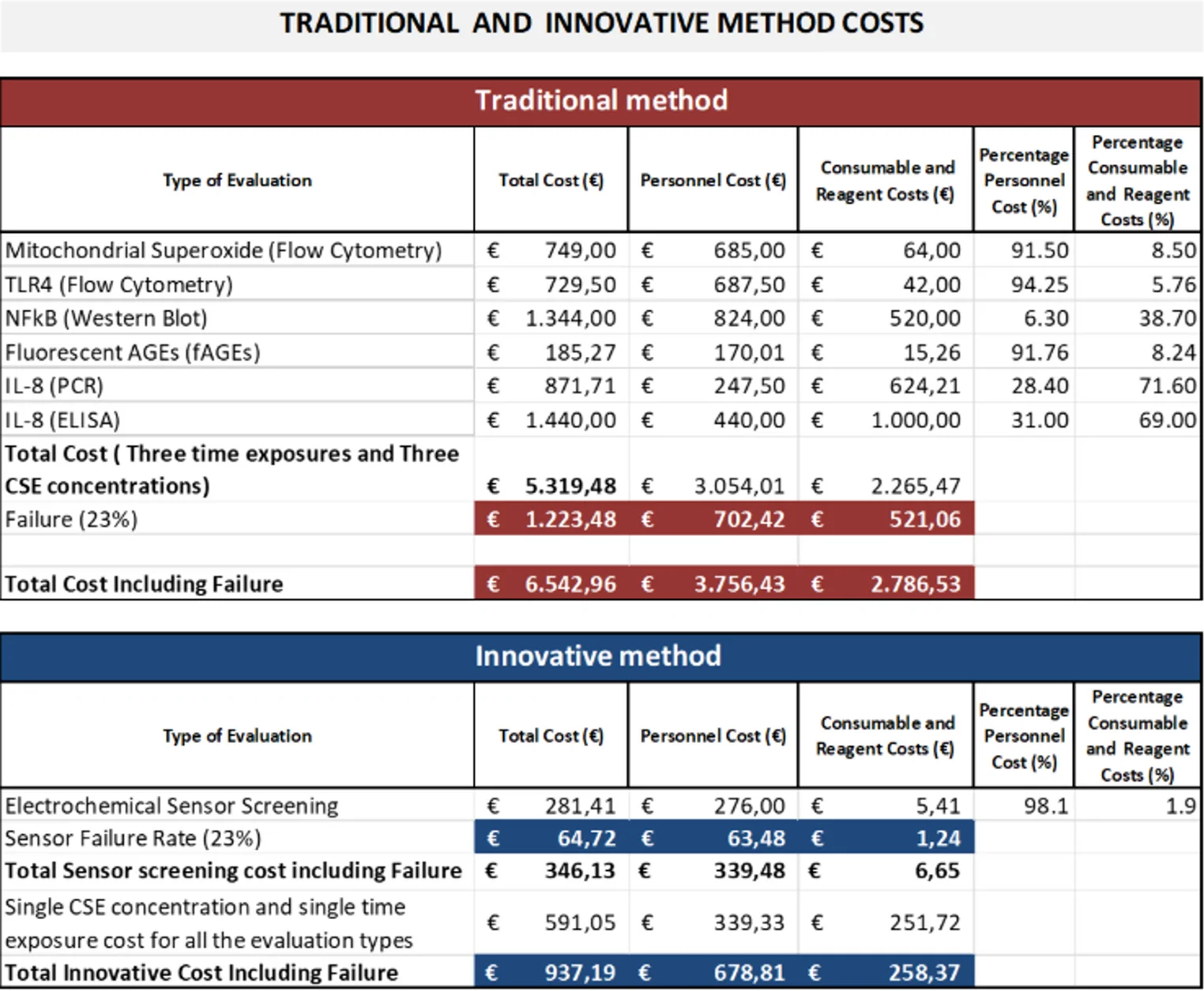
Analytic costs of the traditional and innovative methods

The cost of each experiment was calculated considering exclusively direct costs represented by consumables (plasticware and reagents) and personnel. Indirect costs, which in our Institution correspond to administrative costs (10% of total budget), taxes and costs of utilities and facilities (5% of total budget) were not included in the cost analysis to maintain full comparability across experimental setups. Incorporating these indirect costs would further increase the economic advantage of the innovative sensor-based approach. On the basis of previous published studies [10–12], for each signal, an average number of 5 replicates was considered for obtaining results with a statistically significant relevance. In Fig. 2, the direct analytic costs of both traditional and innovative methods are shown.

Assessment of mitochondrial superoxide production by flow cytometry, considering 4 experimental conditions (NT, CSE 5%, CSE 10% and CSE 20%), *n* = 5 replicates and three time points, has a total cost of 749 euros. The personnel cost is *685* euros (91.5%) and consumables and reagent costs are *64* euros (8.5%).

Evaluation of TLR4 expression by flow cytometry for 4 different experimental conditions, 5 replicates and three time points has a total cost of 729.5 euros. The personnel cost is *687.5* euros (94.24%) and consumables and reagent costs are *42 *euros (5.76%).

Evaluation of NF-kB protein expression by western blot analysis for 4 different experimental conditions, 5 replicates and three time points has a total cost of 1,344 euros. The personnel cost is *824* euros (61.3%) and consumables and reagent costs are *520 *euros (38.7%).

Evaluation of fAGEs for 4 different experimental conditions, 5 replicates and three time points has a total cost of 185.27 euros. The personnel cost is*170.01*euros (91.76%) and consumables and reagent costs are *15.26* euros (8.24%).

Evaluation of IL-8 gene expression by Real Time PCR for 4 different experimental conditions, 5 replicates and three time points has a total cost of 871.71 euros. The personnel cost is *247.5* euros (28.4%) and consumables and reagent costs are *624.21* euros (71.6%).

Evaluation of IL-8 protein release by ELISA for 4 different experimental conditions, 5 replicates and three time points has a total cost of 1,440 euros. The personnel cost is *440*euros (31%) and consumables and reagent costs are *1,000* euros (69%).

In the traditional approach, the baseline cost of all analyses, including flow cytometry, Western blot, Real-Time PCR, and ELISA, totals €5,319.48 that is further increased to 6,542.96 including a 23% (1,223.48) experiment failure applying the following formula on oxidative stress data of a previous study [10]. Number of experiments without oxidative stress induction/total number of experiments x100 based on the data set of previous work.

By contrast, the sensor-based approach incorporates a preliminary, low-cost screening stage at a basic cost of €281.41 (including €5.41 in consumables) to verify whether oxidative stress occurs. Crucially, this early screen identifies non-responding samples before any expensive downstream assays are carried out, confining the regular failure cost to this minimal screening, rather than being applied to the entire experimental budget. This means that the 23% failure rate adds only €64.72 (23% of €281.41), for a total screening expense of €346.13.

At this point, only the cell conditions where oxidative stress is induced require detailed downstream analyses (flow cytometry, Western blot, Real-Time PCR, and ELISA), enabling the selection of both the optimal time point and the most effective concentration of cigarette smoke.

**Fig. 3:**
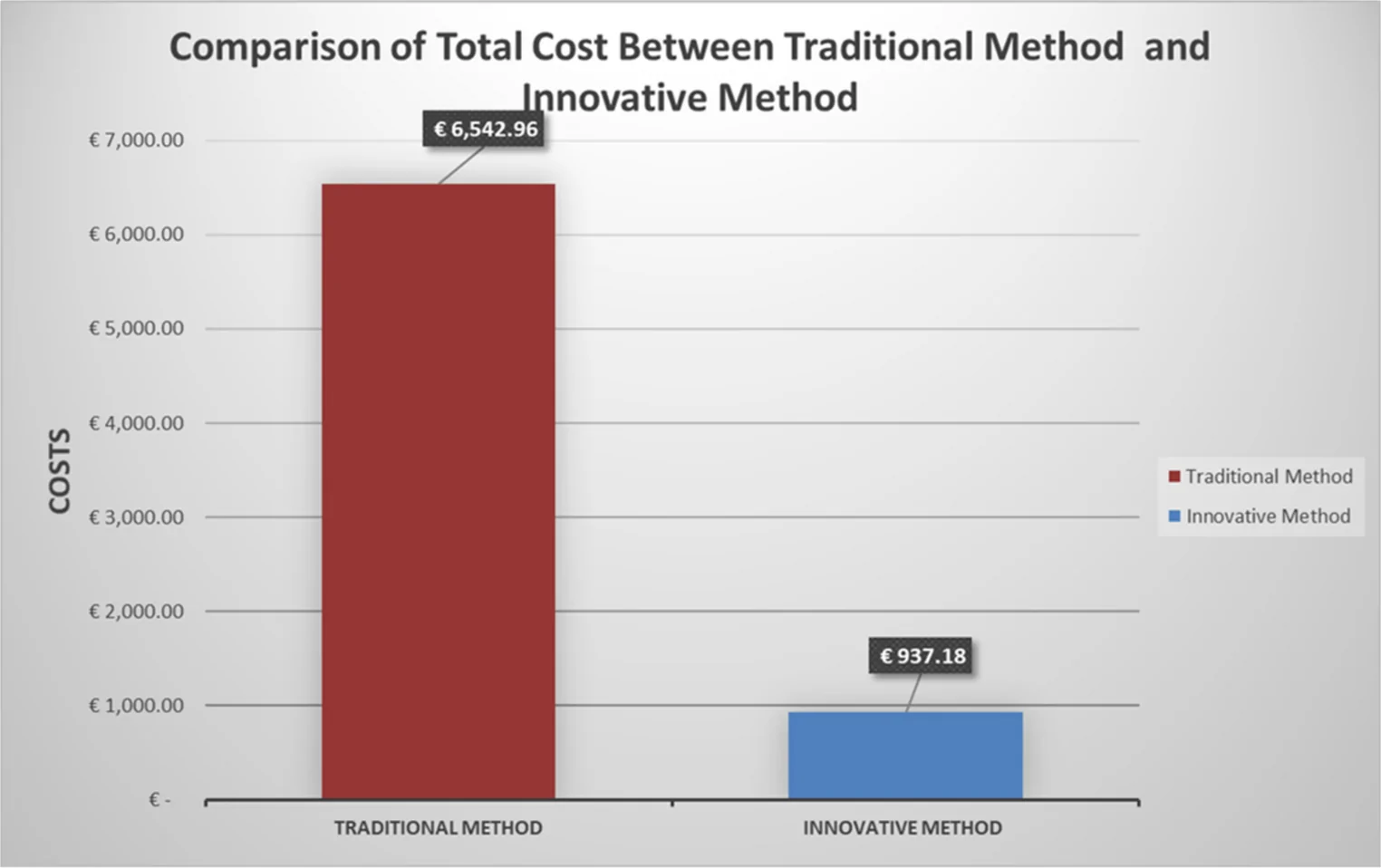
Comparison of total costs between traditional and innovative methods

Consequently, eliminating two of the three planned time points reduces the original analyses to one-third of €5,319.48, or €1,773.16. When €346.13 of screening is added, the total comes to €2,119.29, corresponding to a 67.6% cost reduction compared to the €6,542.96 incurred in the traditional approach (Fig. 3).

Further restricting CSE exposure to the single concentration that maximally induces oxidative stress again divides the €1,773.16 cost by three, resulting in a final cost of €591.05, at which €346.13 is added for screening, totaling €937.18 (Fig. 3). The innovative method total direct cost is 85.67% lower than €6,542.96, underscoring the significant savings achieved by avoiding unnecessary analyses of non-responding samples. As a hypothetical sensitivity scenario, even assuming a 10% technical sensor failure rate, modelled as a corresponding 10% increase in the failure-adjusted screening cost from €346.13 to €380.74, the total estimated costs would be €2,153.90 when selecting a single time point and €971.79 when selecting both a single time point and a single CSE concentration. The estimated cost reductions would remain 67.1% when selecting one time point and 85.1% when selecting both one time point and one CSE concentration.

**Fig. 4:**
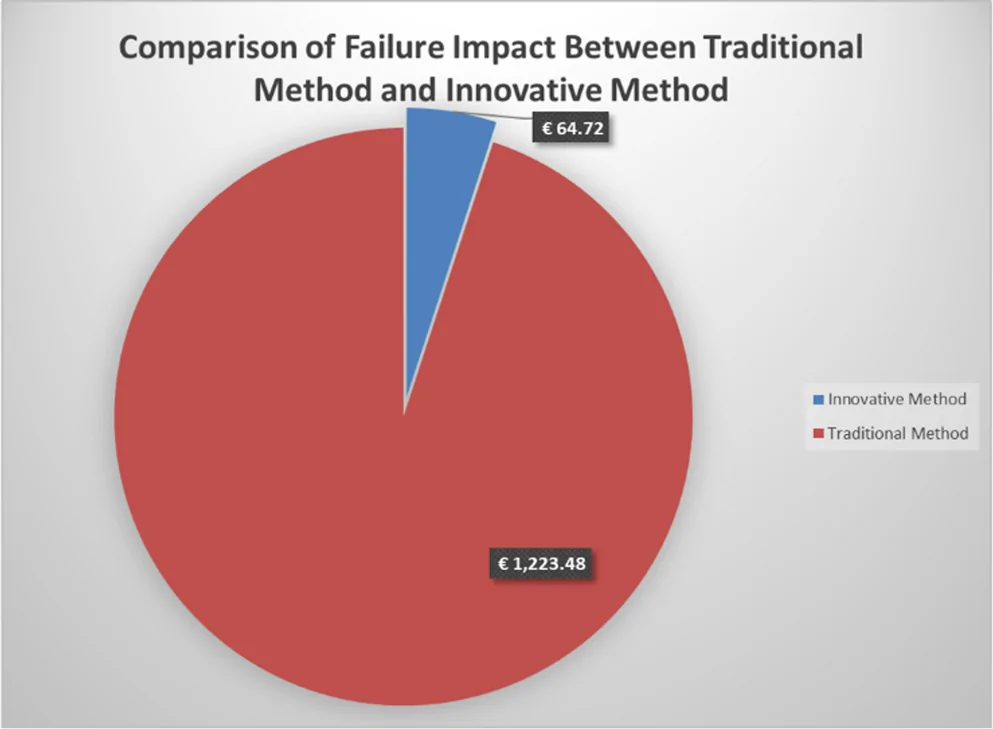
Comparison of failure impact between traditional and innovative methods

Figures 3 and 4 illustrate the economic comparison of total direct costs and the cost of failure of the two methods, respectively.

**Fig. 5:**
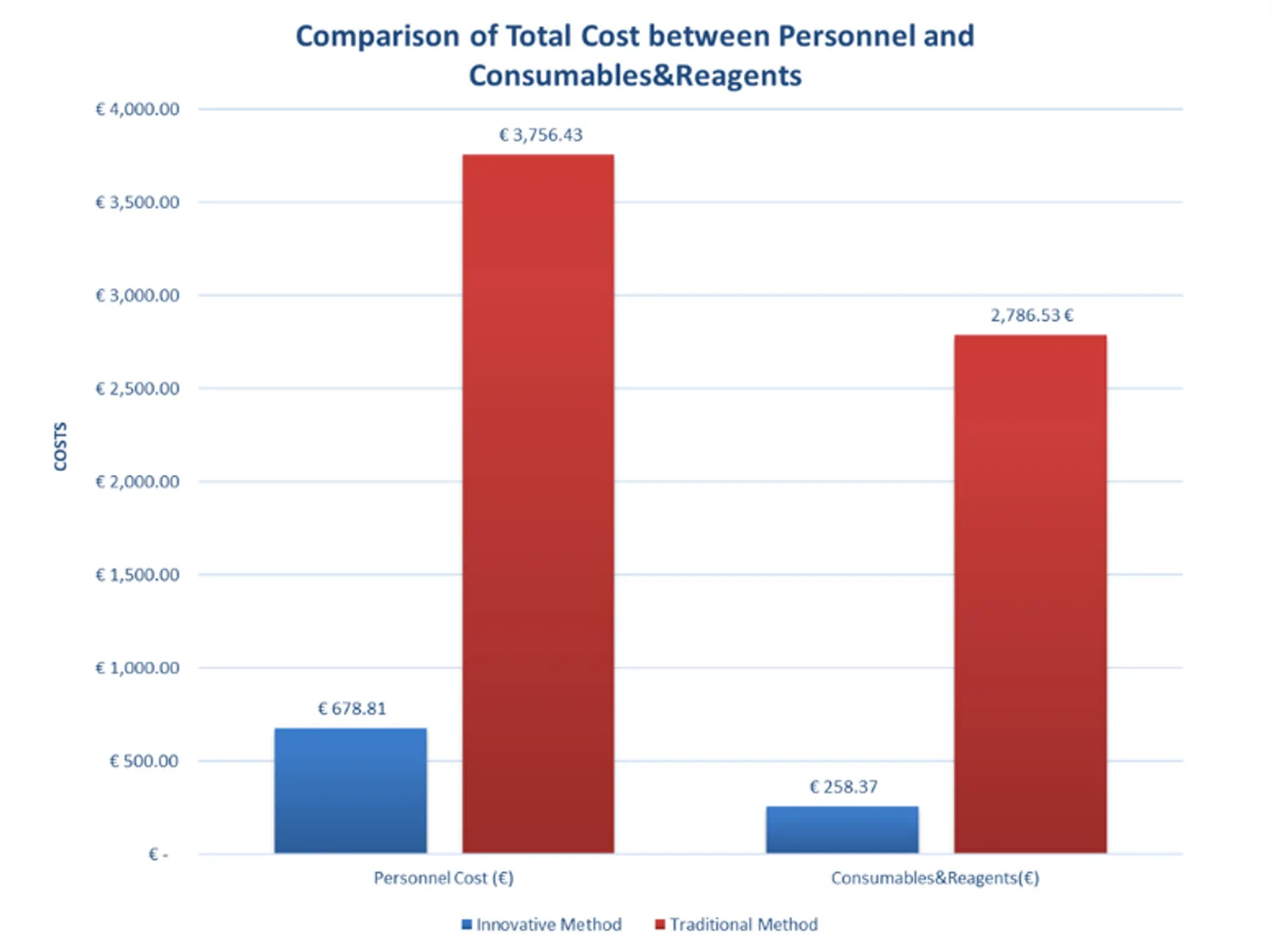
Comparison of personnel, consumable and reagent costs between traditional and Innovative methods

The cost of personnel, consumables and reagents of the traditional method is extraordinarily higher than that of the innovative method (Fig. 5).

**Fig. 6:**
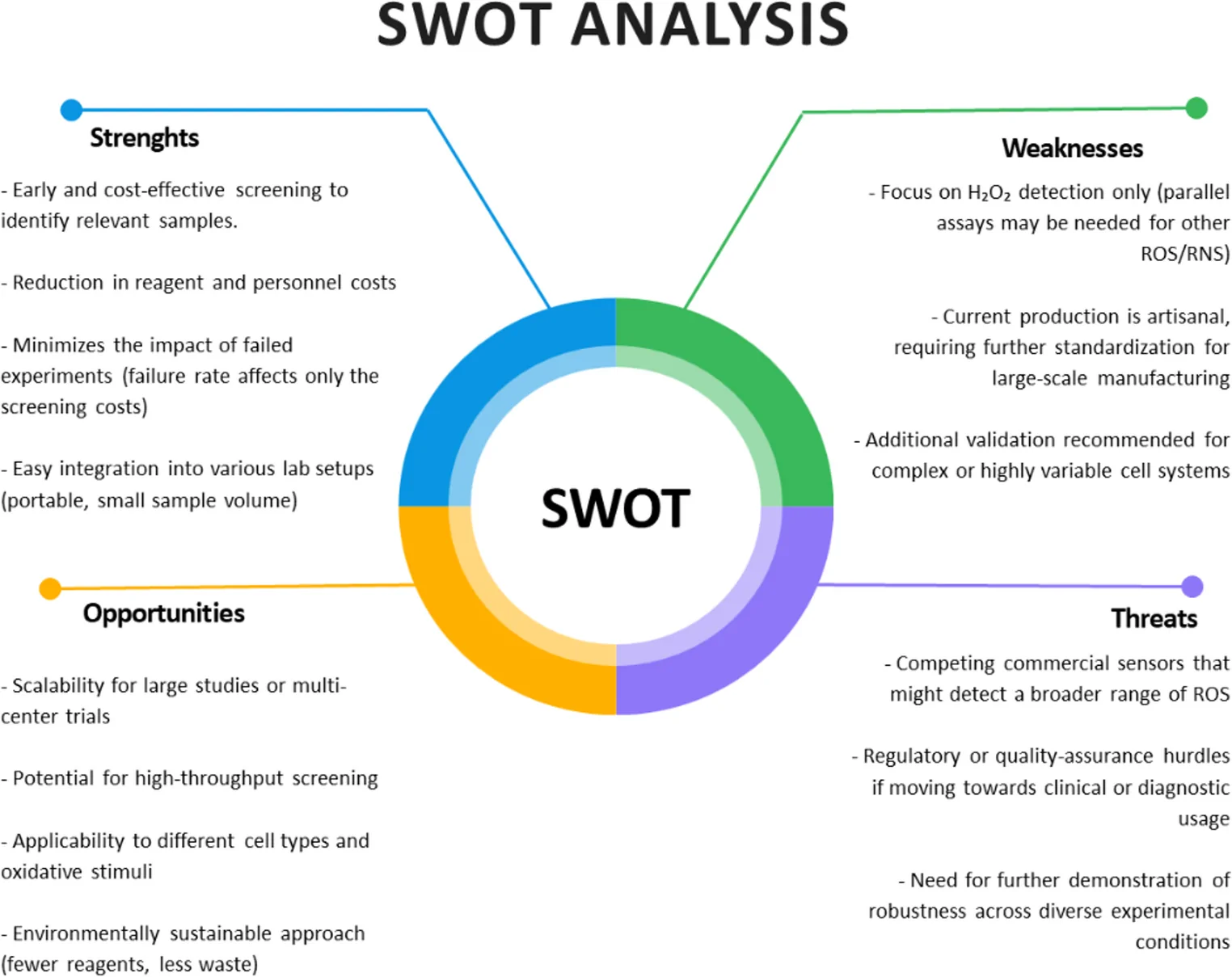
Strengths, weaknesses, opportunities, and threats (SWOT) analysis

To provide a structured overview of this electrochemical approach, we performed *a* SWOT (Strengths, Weaknesses, Opportunities, and Threats) Analysis (Fig. 6).

This framework highlights the primary Strengths, Weaknesses, Opportunities, and Threats associated with our sensor-based methodology for oxidative stress research.

## Discussion

Numerous studies have clarified that aging and the onset of related chronic diseases exhibit a common denominator in the induction of oxidative stress [26, 27]. Oxidative stress originates from the imbalance between the production of free radicals, mainly represented by reactive oxygen species, and the insufficient counterbalancing action offered by antioxidant systems [1]. This scientific evidence on the early pathogenic role played by the induction of oxidative stress in chronic diseases affecting the lung has given impetus to a plethora of studies on techniques and methods to detect oxidative stress and the resulting events. The present study allows a better understanding of the direct costs and supported outputs of a biological study aimed at assessing oxidative stress and the molecular events downstream of increased oxidative stress promoted by smoke exposure by a traditional method and by an innovative approach in a model of airway epithelium. The innovative approach is focused on the use of an electrochemical sensor and has the advantage of screening and then proceeding only those experiments that show increased oxidative stress due to smoke exposure. The innovative approach also allows us to identify efficiently the right time and the right CSE concentration that led to increased oxidative stress in a pre-clinical model of airway epithelial cells. This paper provides detailed descriptions of the methods used to assess the various signals to obtain a more accurate estimate of actual direct costs and to enable the reader to reconstruct the calculations made. To date, the main methods for the detection of this analyte are expensive and time-consuming laboratory techniques such as fluorometric and colorimetric assays. In recent years, electrochemical sensors have been developed and validated as tools that can reliably and reproducibly measure oxidative stress induced by exposure to cigarette smoke in pre-clinical models of airway epithelial cells. Electrochemical sensors are simple to use, can be miniaturized, require small sample volumes (µl), and use very small and portable instrumentation [28]. These sensors may quantify H_2_O_2_ with results comparable to those obtained using classical and established biochemical assays. H₂O₂ was selected as a relatively stable extracellular marker suitable for preliminary screening, but it represents only one component of the overall oxidative response. Other Reactive Oxygen Species (ROS) and Reactive Nitrogen Species (RNS), including superoxide, nitric oxide, and peroxynitrite, require complementary analytical methods. The increase in H₂O₂ measured in the cell culture medium reflected an increase in intracellular oxidative stress assessed by flow cytometry using the mitochondrial superoxide-sensitive fluorescent probe Mitosox Red, which is currently considered the gold standard for intracellular mitochondrial ROS quantification [16–19]. The use of the sensor provides several advantages compared to currently used flow-cytometric or other biochemical approaches. It is faster because it requires only one step (compared to the multiple steps necessary for the flow cytometry approach), and it is a conservative approach because cells remain undisturbed after the removal of the conditioned medium. The sensor is very stable. It is designed as a semi-disposable platform used for one calibration curve and ≤ 80 measurements within the same analytical session. Because each chip is discarded after use, long-term day-to-day drift is not operationally relevant. Analytical performance, therefore, depends on chip-to-chip reproducibility on the day of each experiment, which was ≤ 5%. The sensor also has good selectivity even in the presence of high concentrations of different analytes that could be found in culture media, or that could be generated by cells [17]. The sensor fabrication method is environmentally sustainable and in line with the One-Health context, as it uses recycled waste compact discs in full compliance with circular economy principles [29, 30]. The fabrication workflow (laser-patterning of silver film, deposition of Ag/AgCl and graphite inks, curing, and lamination) mirrors established roll-to-roll and screen-printing processes used for commercial disposable electrochemical strips. These steps can be fully automated, enabling high-volume industrial production with unit costs below €0.50. This paper aims to compare the direct costs of two different approaches for studying oxidative stress in preclinical models: the traditional method and a novel approach based on the use of an electrochemical sensor. This comparison aims to demonstrate the effectiveness of the new approach in reducing direct costs and improving the efficiency of analysis. Although here, the indirect costs applied by our Institution were not included in the evaluation of the costs, this omission does not alter the scope of the paper since adding 15% of indirect costs, the difference between the two methods will be amplified.

As signals evoked by the induction of oxidative stress to be considered in this study, we made use of our previous experimental experiences and literature data established over the years. It is indeed widely known that cigarette smoke exposure of bronchial epithelial cells induces oxidative stress [31], resulting in altered gene and protein expression of receptors for innate immunity such as TLR4 and in increased release of potent inflammatory cytokines such as IL-8 [12, 21]. We have further planned to include fAGEs assessment because increased levels are linked to increased extracellular oxidative stress [14] and because our recent data demonstrate a positive and significant correlation (Rho: 0.548; *p* = 0.0044 by Spearman test) between the levels of hydrogen peroxide assessed by the electrochemical sensor and levels of fAGE [20]. Here, we newly performed retrospective analyses of some data from a previous work [20] obtained by 16HBE not treated (*n* = 5) and stimulated with CSE (*n* = 5) and CSE+CARBO *n* = 5 and then analyzed by the electrochemical sensor, Mitosox assay, and fAGE fluorimetry. We showed positive and significant correlations between Mitosox and electrochemical sensor data (Rho 0.6; *p* = 0.023 by Spearman test) and between data obtained by Mitosox assay and fAGE fluorimetry (Rho 0.82; *p* = 0.0019 by Spearman test). All together these findings further support the reliability of the sensor here under study. Table 1 provides an overview of the main performance indicators of the three different methods here used for evaluating oxidative stress.

**Table 1:** Comparison of the main performance indicators of the techniques used for assessing oxidative stress

Method	Analyte measured	Analytical principle	LOD	Quantification	Assay time	Sample volume	Equipment required
Innovative electrochemical sensor	H_2_O_2_	Voltammetric detection	60 µM	100–500 µM	< 1 min	< 1 mL	Portable potentiostat
Flow cytometry	Mitochondrial O₂⁻	Fluorescent probe	5–20 nM	Limited by probe variability	30–60 min	500 µL	Flow cytometer, plate reader
Fluorescence spectroscopy	fAGEs	Fluorescent intensity measurement (emission at 440 nm and excitation at 350 nm)	-	Arbitrary units/protein concentration	10–30 min	50–200 µL	Plate reader

Preclinical cellular models of ageing represent a key tool that will foster future clinical translation application and future changing clinical practice for advanced and early diagnosis or for the establishment of new treatment efficacy [4, 32, 33].The innovative approach with the electrochemical sensor allows early identification of experiments in which oxidative stress does not occur, avoiding the need to continue with costly analyses on irrelevant samples or due to inadequate stimulus concentrations or exposure times. This method improves operational efficiency and reduces overall research costs. For the innovative method with the electrochemical sensor, the concept of failure applies to preliminary screening. However, the approach makes it possible to identify immediately whether oxidative stress has been induced or not, avoiding the need to continue with expensive experiments on irrelevant samples. The electrochemical sensor immediately filters out ineffective experiments, so the cost of failure does not affect subsequent complex analysis experiments. The failure rate considered is still 23%, but this only applies to the cost of the preliminary screening (€281.41) and not to the whole process. The results of the present study provide a process innovation in biomedical studies aimed at assessing oxidative stress and downstream events. The innovation process relies on a new method for finding a more efficient way to operate, and a new driving force behind sustainable growth and competitive advantage. The optimization of the number of experiments useful to obtain statistically relevant results leads to reduced waste production, thus obtaining better environmental preservation. The reduced consumable and reagent costs, as well as the reduced human capital costs, allow the same money and time to carry on more than five projects. While our results support the advantages of this sensor-based method, several limitations warrant mention. First, because the system is optimized primarily for H₂O₂ detection, more complex protocols may still require additional assays to capture other facets of oxidative or nitrosative stress. Second, the sensor fabrication process, being largely “homemade,” might require further standardization or commercial upscaling to ensure consistent reproducibility at an industrial scale. This can be easily obtained using industrial automation and quality-control systems. Materials (silver film, conductive inks) and processes (laser cutting, screen-printing, curing) are already widely standardized for commercial electrochemical sensors, supporting scalable and reproducible production. Finally, although we have demonstrated significant cost savings in our scenario, broader adoption across multiple laboratories would benefit from inter-laboratory validation and further refinement of the sensor design. In addition, the base-case model assumes proportional scaling of condition-dependent costs, whereas some fixed components, such as shared controls, calibration standards, and bulk reagent preparation, may not decrease proportionally. However, the hypothetical sensitivity scenario showed that the estimated economic advantage remained substantially unchanged even when a 10% technical sensor failure rate was assumed. Nonetheless, these limitations do not diminish the potential benefits of the approach. Future studies may explore integrating this screening tool with other technologies or expanding to additional cell lines and disease models. The ability to rapidly screen for oxidative stress before pursuing detailed molecular assays can be a critical advantage, especially in large-scale or resource-constrained research environments.

In conclusion, economic analyses of biomedical studies are increasingly needed in the evaluation and introduction of new technologies. Researchers are expected to consider the cost-effectiveness of the innovative interventions that they choose, and an understanding of economic evaluations is also essential for readers to critically review the available literature of technological innovations. The electrochemical sensor-based screening stands out as an effective and economical strategy for oxidative stress studies, offering early identification of pertinent samples and reducing the burden of downstream analyses. Coupled with its scalability and portability, this method holds promise for a wide range of applications, from basic research to multicenter translational studies.

By integrating a succinct cost-benefit perspective and the SWOT framework, we illustrate how process innovation in biomedical research can advance both sustainability and scientific rigor. Further improvements in sensor design and broader validation are likely to reinforce its role as a rapid, cost-effective alternative to conventional oxidative stress assessment methods.

## Data Availability

The data used to support the findings of this study are included within the article.

## Abbreviations

ROS: Reactive Oxygen Species
RNS: Reactive Nitrogen Species
CSE: Cigarette Smoke Extract
LOD: Limit Of Detection
WE: Working Electrode
CE: Counter Electrode
CD: Compact Discs
LSV: Linear Sweep Voltammetry
AU: Arbitrary Units
MEM: Minimum Essential Medium
TLR4: Toll Like Receptor 4
COPD: Chronic Obstructive Pulmonary Disease
